# F-box and WD repeat domain containing 7 inhibits the activation of hepatic stellate cells by degrading delta-like ligand 1 to block Notch signaling pathway

**DOI:** 10.1515/med-2023-0634

**Published:** 2023-04-17

**Authors:** Yufeng Sun, Lili He, Peiran Guo, Fenghua Li, Bo Wang, Yifan Zhang, Kai An, Ming Peng

**Affiliations:** Department of Gastroenterology, The Second Hospital of Hebei Medical University, No. 215, West Heping Road, Shijiazhuang, Hebei, 050000, China; Department of Emergency, Hebei Provincial Hospital of Traditional Chinese Medicine, Shijiazhuang, Hebei, China; College of Integrated Traditional Chinese and Western Medicine, Hebei Medical University, Shijiazhuang, Hebei, China; Department of Integrated Traditional Chinese and Western Medicine, Shijiazhuang First Hospital, Shijiazhuang, Hebei, China; Department of Rehabilitation, Shijiazhuang First Hospital, Shijiazhuang, Hebei, China; The Second Hospital of Hebei Medical University, Shijiazhuang, Hebei, China

**Keywords:** hepatic fibrosis, LX-2 cells, delta-like ligand 1, ubiquitination, F-box and WD repeat domain containing 7

## Abstract

Hepatic fibrosis (HF) is a precursor of liver cirrhosis, and activated hepatic stellate cells are an important driver of fibrosis. F-box and WD repeat domain containing 7 (FBXW7) expression level is down-regulated in HF, but the underlying mechanism is yet to be elucidated. The interaction between FBXW7 and delta-like ligand 1 (DLL1) was predicted. LX-2 cells were subjected to transfection of FBXW7/DLL1 silencing or overexpression plasmid. The expressions of FBXW7 and DLL1 in HF *in vitro* were measured by quantitative reverse transcription polymerase chain reaction and western blot. The LX-2 cell cycle, viability, proliferation, and ubiquitination were determined by flow cytometry, cell counting kit-8, colony formation, and ubiquitination assays, respectively. FBXW7 overexpression suppressed the cell viability and proliferation, facilitated cell cycle arrest, and down-regulated α-smooth muscle actin (α-SMA), Collagen I, and DLL1 protein levels, but FBXW7 silencing did the opposite. DLL1 was bound to and ubiquitin-dependently degraded by FBXW7 overexpression. DLL1 overexpression promoted the cell viability and proliferation, accelerated cell cycle, and up-regulated the levels of α-SMA, Collagen I, NOTCH2, NOTCH3, and HES1, but these trends were reversed by FBXW7 overexpression. To sum up, FBXW7 overexpression suppresses the progression of HF *in vitro* by ubiquitin-dependently degrading DLL1.

## 1 Introduction

Hepatic fibrosis (HF) is a cause of increasing mortality around the world. At present, the incidence of HF still presents an increasing trend owing to the growth and aging of the population, unhealthy diet, as well as the epidemic of nonalcoholic fatty liver disease [1–3]. HF is the ultimate pathology of various chronic diseases such as chronic viral hepatitis and non-alcoholic fatty liver disease [4]. In the development of HF, hepatic stellate cells (HSCs) enter an activated state, and produce excessive collagen-based extracellular matrix (ECM) such as α-smooth muscle actin (α-SMA), thereby leading to the collagen deposition and inducing tissue fiberization, which is the key to the alteration in the normal structure and function of the liver tissues [5–7]. Patients with HF do not manifest obvious clinical symptoms in the early stage, but they may progress to liver cirrhosis or even liver cancer in 20–40 years [8,9]. Hence, it is of great importance to deepen the understanding of HSC activation.

The development of HF involves various signaling pathways and multiple cytokines [10,11]. Notably, JAK/STAT, NF-κB, and Notch signaling pathways have been identified to be closely associated with HSC activation, among which Notch signaling pathway can be regulated by four Notch receptors and ligands (delta-like ligand 1 [DLL1], DLL3, DLL4, Jagged 1, and Jagged 2), playing an important role in homeostatic processes [12–15].

With the in-depth understanding of epigenetics, post-translational modifications such as ubiquitination have been confirmed to be an important mechanism of disease occurrence [16]. As we know, ubiquitin-proteasome system-driven ubiquitination is the main pathway for intracellular protein degradation, which has been increasingly found in the development of HF [17,18]. During the process of ubiquitination, ubiquitin ligase (E3) plays a pivotal role in the identification of target proteins and subsequent protein degradation [19]. Through the prediction of Ubibrowser, we found six ubiquitylases that are highly bound to DLL1, including F-box and WD repeat domain containing 7 (FBXW7). It is worth noting that FBXW7, one member of E3 family, has been reported to express at a low level in HF *in vivo* [20]. In line with the above-mentioned information, it is reasonable to speculate that FBXW7 may alleviate HF by reducing DLL1 level. Initially, FBXW7 has been considered as a tumor suppressor, and there is growing evidence showing that FBXW7-mediated ubiquitination is able to exert regulatory effects on cell cycle, proliferation, differentiation, and apoptosis [21–24]. However, the regulatory effect of FBXW7-mediated ubiquitination on HSC activation in HF is poorly understood.

Herein, we determined to decipher the effect of FBXW7 on HSC activation and its possible mechanism in HF. Based on our present findings, it is indicative that targeting FBXW7 could be a potential target for HF treatment.

## 2 Materials and methods

### 2.1 Bioinformatics analysis

The Ubibrowser (http://ubibrowser.bio-it.cn/ubibrowser_v3/) was applied to predict putative E3 ligases of DLL1.

### 2.2 Cell culture and transfection

Human HSCs LX-2 purchased from Procell (CL-0560; Wuhan, China) were cultured in Dulbecco’s Modified Eagle’s Medium (DMEM) containing 10% FBS and 1% penicillin–streptomycin (10,000 units/mL penicillin and 10,000 μg/mL streptomycin) (15140122; Gibco, Carlsbad, CA, USA) in a 5% CO_2_ incubator (Forma™ II 3110; Thermo Scientific, Waltham, MA, USA) at 37°C. Overexpression plasmids of FBXW7 and DLL1 as well as their negative control (NC) were obtained from Sino Biological (HG29610-UT, HG11635-UT, CV011; Beijing, China). Short hairpin RNA against FBXW7 (shFBXW7) and its NC (shNC) were synthesized by GenePharma (Shanghai, China). Lipofectamine 2000 Transfection Reagent (11668030; Invitrogen, Carlsbad, CA, USA) was dissolved in DMEM. The above plasmids, NC, shFBXW7, or shNC were treated with Lipofectamine solution according to the protocol. The mixed solution was added into a 96-well plate to treat LX-2 cells for 4 h in the incubator until 70–90% confluence was reached. Afterward, the solution was replaced with culture medium for another 48 h culture.

### 2.3 Cell counting kit-8 (CCK-8) assay and colony formation assay

LX-2 cells were seeded in 96-well plates at the density of 5 × 10^3^ cells/well after 48 h of transfection. At 0, 24, 48, and 72 h, the culture medium was discarded. Then, CCK-8 solution (C0037; Beyotime, Suzhou, China) diluted with DMEM at a ratio of 1:9 was added into each well to treat the cells for 2 h. A microplate reader (E1140; Beyotime, China) was used to determine the absorbance at 490 nm.

Two hundred transfected LX-2 cells were seeded in a 75 mm plate (Corning, Corning, NY, USA) and cultured for 14 days. The serum-free medium was replaced every 4 days. After removal of the culture medium, PBS was used to wash the cells three times. Thereafter, the cells were added with 4% paraformaldehyde (P1110; Solarbio, Beijing, China) for 15 min, and subsequently stained with Giemsa solution (G1015; Solarbio, China). Finally, the cell colonies were observed using an Eclipse 80i microscope (Nikon, Tokyo, Japan).

### 2.4 Cell cycle detection

After 48 h of transfection, LX-2 cells were subjected to detection of cell cycle using the DNA Content Quantitation Assay Kit (CA1510; Solarbio, China). Concretely, the cells were digested by trypsin (EDTA 0.25%) (25200056; Gibco, USA), and resuspended with DMEM. Then 70% ethanol (48075; Merck, Darmstadt, Germany) was added to treat the cells at 4°C for 12 h, followed by washing with pre-cooled PBS. Subsequently, the cells (1 × 10^6^ cell/mL) were reacted with 100 µL RNase solution in a bath at 37°C for 30 min, and then were mixed with 400 µL propidium iodide solution at 4°C in the dark. After 30 min, the cell cycle of the cells was analyzed by a flow cytometer (CytoFLEX, Beckman Coulter, Brea, CA, USA) at an excitation wavelength of 488 nm.

### 2.5 Ubiquitination assay

For co-immunoprecipitation, 293T cells transfected with Flag‐FBXW7 and HA-DLL1 recombinant plasmids were homogenized in NP-40 Lysis Buffer (P0013F; Beyotime, Shanghai, China) containing MG-132 (S1748; Beyotime, China) and deubiquitinase inhibitor with anti‐Flag (F7425, 1:1,500; Sigma, USA) or anti‐HA (51064‐2‐AP, 1:1,000; Proteintech, USA) antibody at 4°C overnight, followed by coupling with A/G agarose beads for 4 h. Afterward, the immunoprecipitates were subjected to the detection of western blot again.

### 2.6 Quantitative reverse transcription polymerase chain reaction (qRT-PCR)

Total RNAs from LX-2 cells transfected with or without silencing/overexpression plasmid of FBXW7 and/or DLL1 were isolated using the RNA isolation kit (AM1912; Invitrogen, USA). After RNA quantitation, complementary DNAs (cDNAs) were synthesized from the extracted RNAs using a cDNA synthesis kit (18090010; Invitrogen, USA) according to the instructions. With addition of PCR Master Mix (FPCR-RO, Sigma-Aldrich, St Louis, MO, USA) and specific PCR primers, cDNA amplification was performed according to the reaction condition below: 45 cycles of annealing at 95°C for 15 s, followed by 55°C for 60 s and 72°C for 60 s. The relative mRNA levels were calculated using the 2^−ΔΔCt^ method [25], and then normalized against GAPDH. All sequences of primers in this reaction are as follows (5′–3′): FBXW7 (GTGAGAGAACGCTGAGGAGG, GGGACAGTCAGGTTTGGGAG), α-SMA (CTGTTCCAGCCATCCTTCAT, CGGCTTCATCGTATTCCTGT), Collagen I (GATTCCCTGGACCTAAAGGTGC, AGCCTCTCCATCTTTGCCAGCA), DLL1 (CTCGGTGATGCCTACCTGTG, ACACTCGCACACATAGCGGT), and GAPDH (TCAAGGCTGAGAACGGGAAG, TGGACTCCACGACGTACTCA).

### 2.7 Western blot

Total proteins from LX-2 cells with or without transfection of FBXW7 and/or DLL1 silencing or overexpression plasmid were lysed using extraction buffer (89901; Thermo Fisher, USA). After centrifugation (12,000 rpm, 4°C), cell supernatant was subjected to protein quantitation using the BCA kit (23225; Thermo Fisher, USA) in light of the protocol. Equal amounts of proteins (20 μg) were separated by 6–10% sodium dodecyl sulfate-polyacrylamide gel electrophoresis, and transferred to polyvinylidene difluoride membranes (03010040001; Sigma-Aldrich, USA). Then, the membranes were stained using Ponceau solution (P0012, Solarbio, China). The blank sites of the membranes were blocked by 5% BSA solution (SW3015, Solarbio, China). Thereafter, the membranes were incubated with primary antibodies (Abcam, Cambridge, UK) against α-SMA (ab5694, 1:1,000, 42 kDa), Collagen I (ab34710, 1:1,000, 130 kDa), DLL1 (ab10554, 1:1,000, 78 kDa), NOTCH2 (ab118824, 1:500, 265 kDa), NOTCH3 (ab252845, 1:1,000, 244 kDa), Hairy and Enhancer of Split 1 (HES1; ab119776, 1:500, 130 kDa), FBXW7 (55290-1-AP; 1:1,000, 80 kDa Proteintech, Millipore, USA), and GAPDH (ab8245, 1:10,000, 36 kDa) at 4°C for 16 h, followed by 2 h incubation with secondary antibodies (ab7090, ab6728, ab6734; Abcam, UK) at 37°C. Afterward, band densities were visualized using an ECL kit (CPS160; Sigma-Aldrich, USA) and a chemiluminescence imaging system (5200 Multi, Tanon, Shanghai, China).

### 2.8 Statistical analysis

The above experiments were independently repeated at least three times. All data were expressed as mean ± standard deviation and analyzed using SPSS software (version 22.0, IBM, Armonk, NY, USA). Differences among multi-groups were analyzed by one-way analysis of variance, followed by Tukey’s *post hoc* test. *p* < 0.05 was considered to be statistically significant.

## 3 Results

### 3.1 FBXW7 served as a possible E3 ligase of DLL1

**Figure 1:**
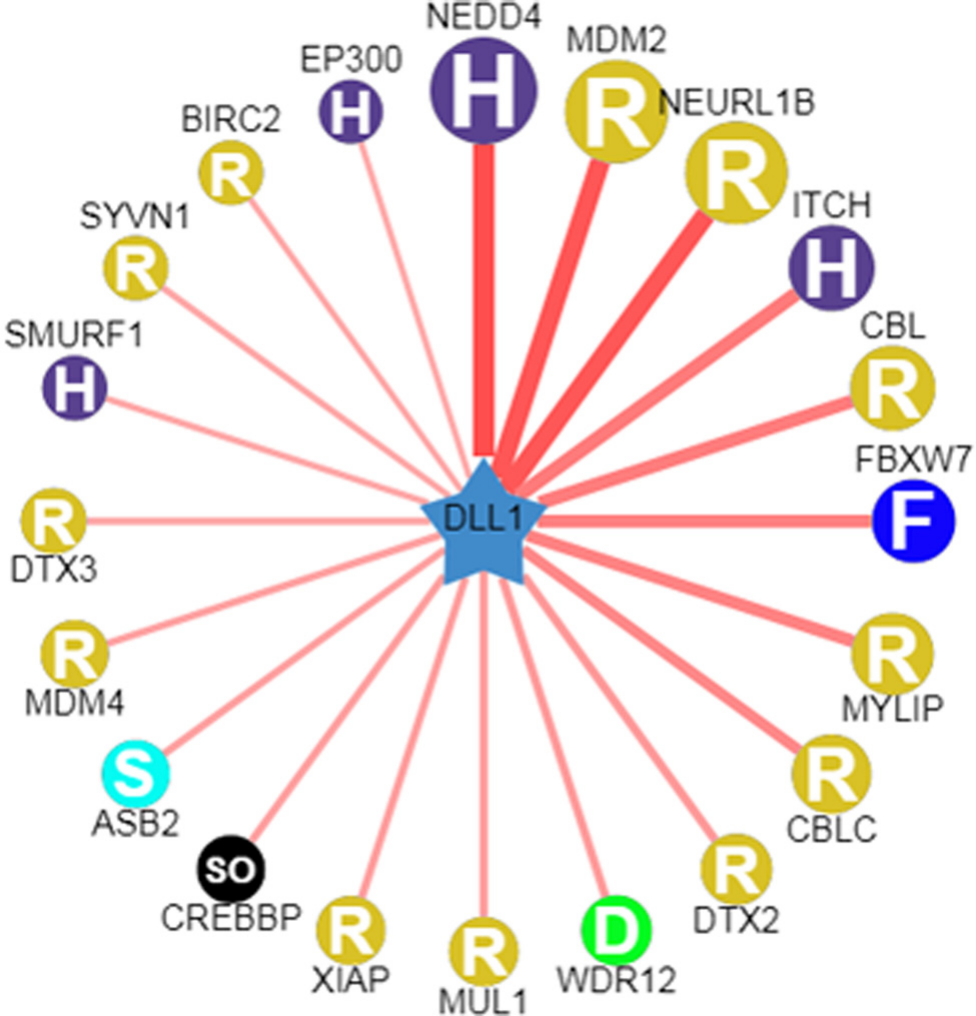
Bioinformatics prediction of DLL1. The Ubibrowser (http://ubibrowser.bio-it.cn/ubibrowser_v3/) was applied to predict putative E3 ligases of DLL1. E3, ubiquitin-ligase enzyme; DLL1, delta-like ligand 1; H, HECT (homologous to the E6-AP carboxyl terminus); R, RING (really interesting new gene); F, F-box; D, DWD; S, SOCS-box; SO, single other. The predicted interactors are arranged clockwise in a descending order according to the confidence score. The width of the red edge reflects the confidence of the interaction.

As illustrated in Figure 1, bioinformatics prediction identified six E3 ligases (NEDD4, MDM2, NEURL1B, ITCH, CBL, and FBXW7) with high binding potential to DLL1. Given that FBXW7 is lowly expressed in HF-modeled animals [20], we thereby selected FBXW7 as the subject gene in this study.

### 3.2 FBXW7 regulated the viability and proliferation of LX-2 cells

**Figure 2:**
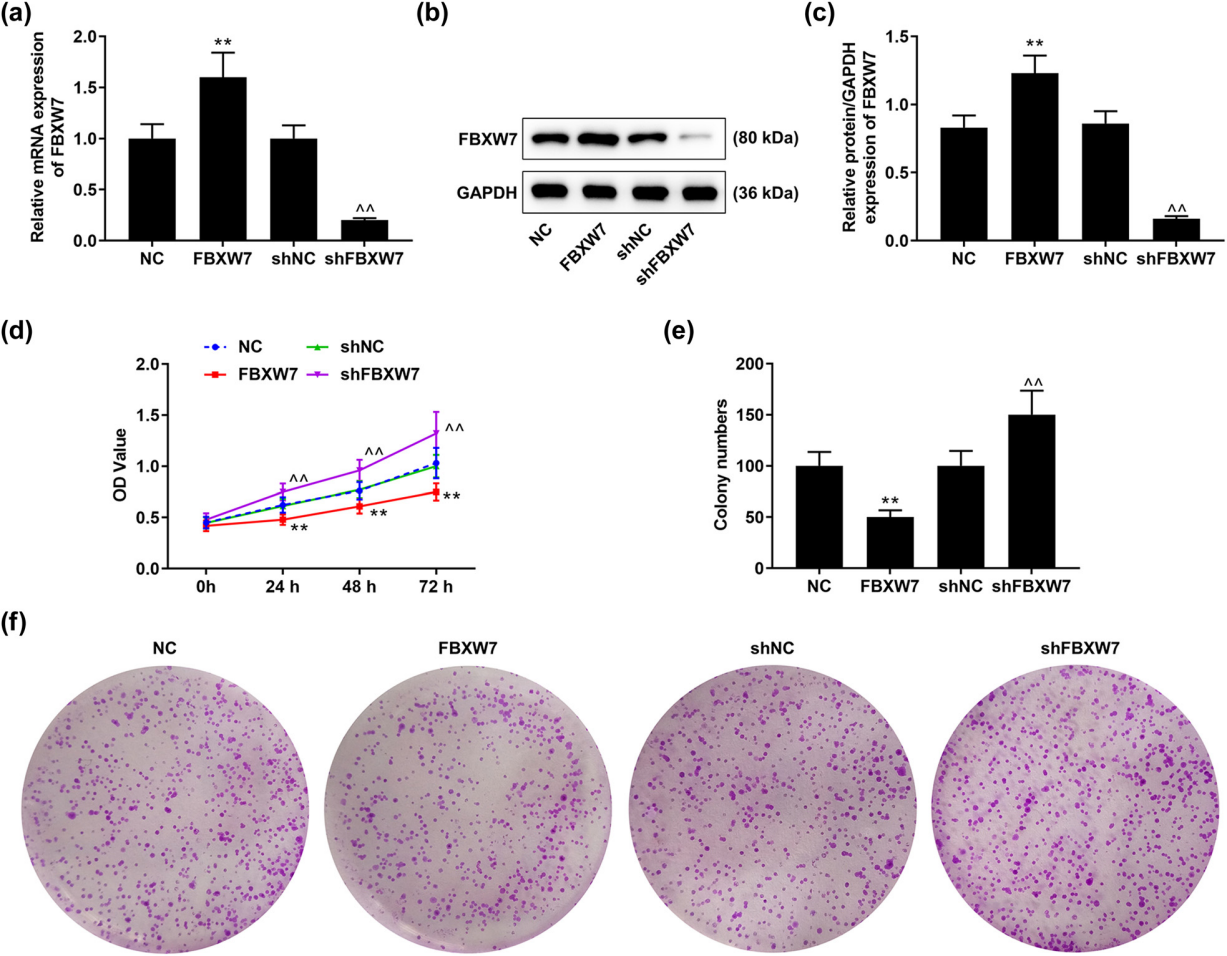
Effects of FBXW7 on LX-2 cell viability and proliferation. LX-2 cells were transfected with or without FBXW7 silencing or overexpression plasmid. The expression of FBXW7 in the transfected cells was determined by qRT-PCR and western blot, with GAPDH serving as the internal control (a, b and c). LX-2 cell viability at 0, 24, 48, and 72 h was analyzed by CCK-8 assay (d). Colony formation assay was used to detect the colony number of LX-2 cells (e and f). The significant difference was measured by ANOVA, followed by Tukey’s *post hoc* test. ^**^ *p* < 0.001 vs NC; ^^^^ *p* < 0.001 vs shNC. ANOVA, analysis of variance; CCK-8, cell counting kit-8; FBXW7, F-box and WD repeat domain containing 7; h, hour; shFBXW7, short hairpin RNA against FBXW7; shNC, shRNA negative control; qRT-PCR, quantitative reverse transcription polymerase chain reaction.

To further understand the role of FBXW7 in HF, the expression of FBXW7 was interfered via cell transfection. As shown in Figure 2a and b, FBXW7 expression was up-regulated by FBXW7 overexpression plasmid, but was down-regulated by shFBXW7 in LX-2 cells (*p* < 0.001). Through CCK-8 assay, we observed that the viability of LX-2 cells was weakened in the presence of FBXW7, but was strengthened in the absence of FBXW7 (Figure 2c, *p* < 0.001). Similarly, FBXW7 overexpression induced a decline of LX-2 cell viability and proliferation, but FBXW7 silencing did the opposite (Figure 2d, e and f, *p* < 0.001).

### 3.3 FBXW7 regulated cell cycle as well as α-SMA and Collagen I expressions in LX-2 cells

**Figure 3:**
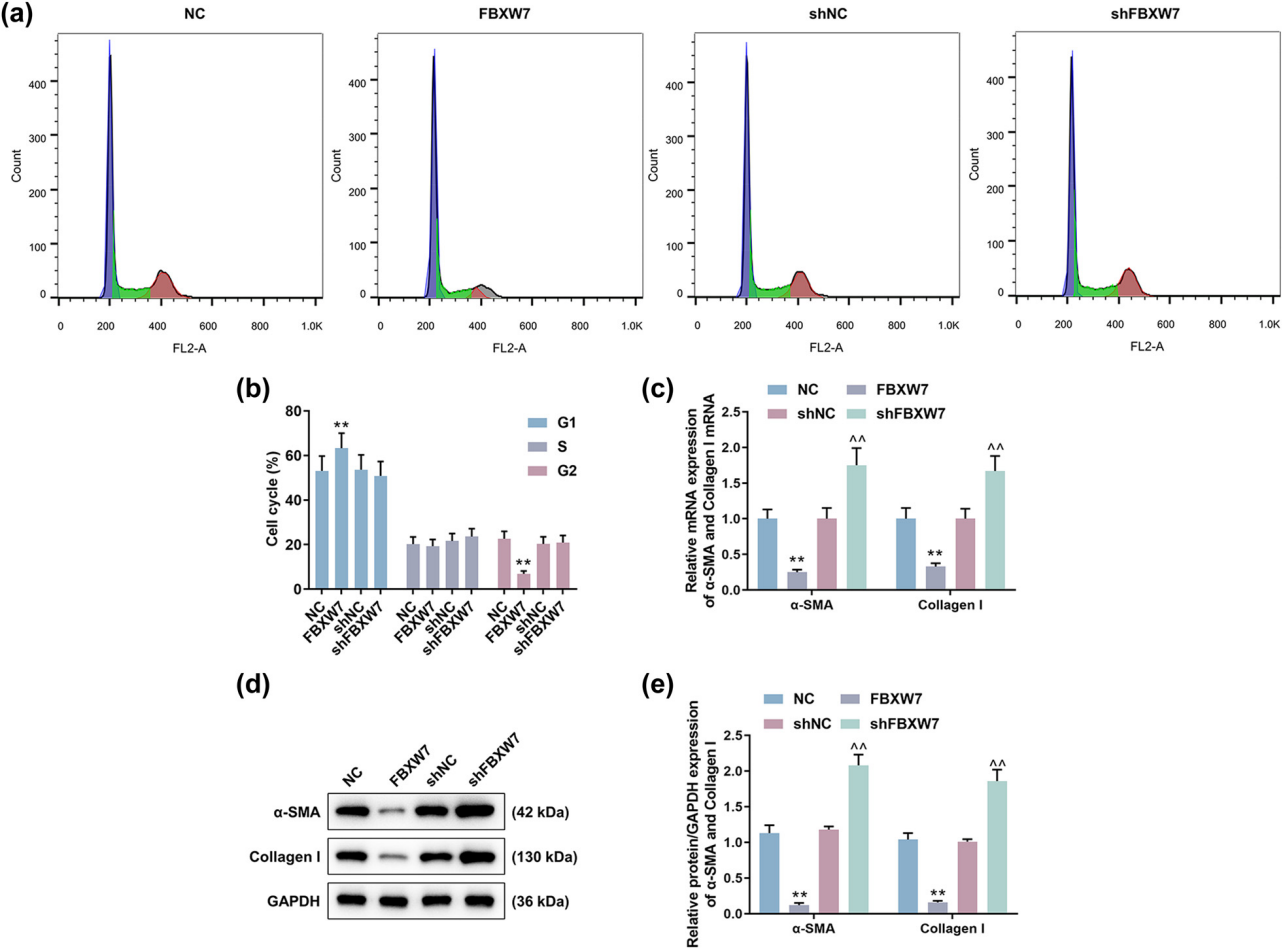
Effects of FBXW7 on cell cycle and the levels of α-SMA and Collagen Ⅰ in LX-2 cells. LX-2 cells were transfected with or without FBXW7 silencing or overexpression plasmid. A cell cycle kit and flow cytometry were applied for the determination of cell cycle (a and b). The expressions of α-SMA and Collagen Ⅰ in LX-2 cells were measured by qRT-PCR (c) and western blot (d and e), where GAPDH was used as the internal control. The significant difference was determined by ANOVA, followed by Tukey’s *post hoc* test. ^**^ *p* < 0.01 vs NC; ^^^^ *p* < 0.01 vs shNC. ANOVA, analysis of variance; FBXW7, F-box and WD repeat domain containing 7; shFBXW7, short hairpin RNA against FBXW7; shNC, shRNA negative control; qRT-PCR, quantitative reverse transcription polymerase chain reaction.

According to the results of detection on cell cycle, we discovered that FBXW7 overexpression evidently extended the G1 phase yet shortened the G2 phase of LX-2 cells, and no significant change was observed in cell cycle after FBXW7 silencing (Figure 3a and b, *p* < 0.001). As demonstrated in Figure 3c–e, α-SMA and Collagen Ⅰ expressions in LX-2 cells were reduced in the presence of FBXW7 (*p* < 0.01), but were elevated in the absence of FBXW7 (*p* < 0.01).

### 3.4 DLL1 was degraded by FBXW7 in LX-2 cells through an ubiquitin-dependent way

**Figure 4:**
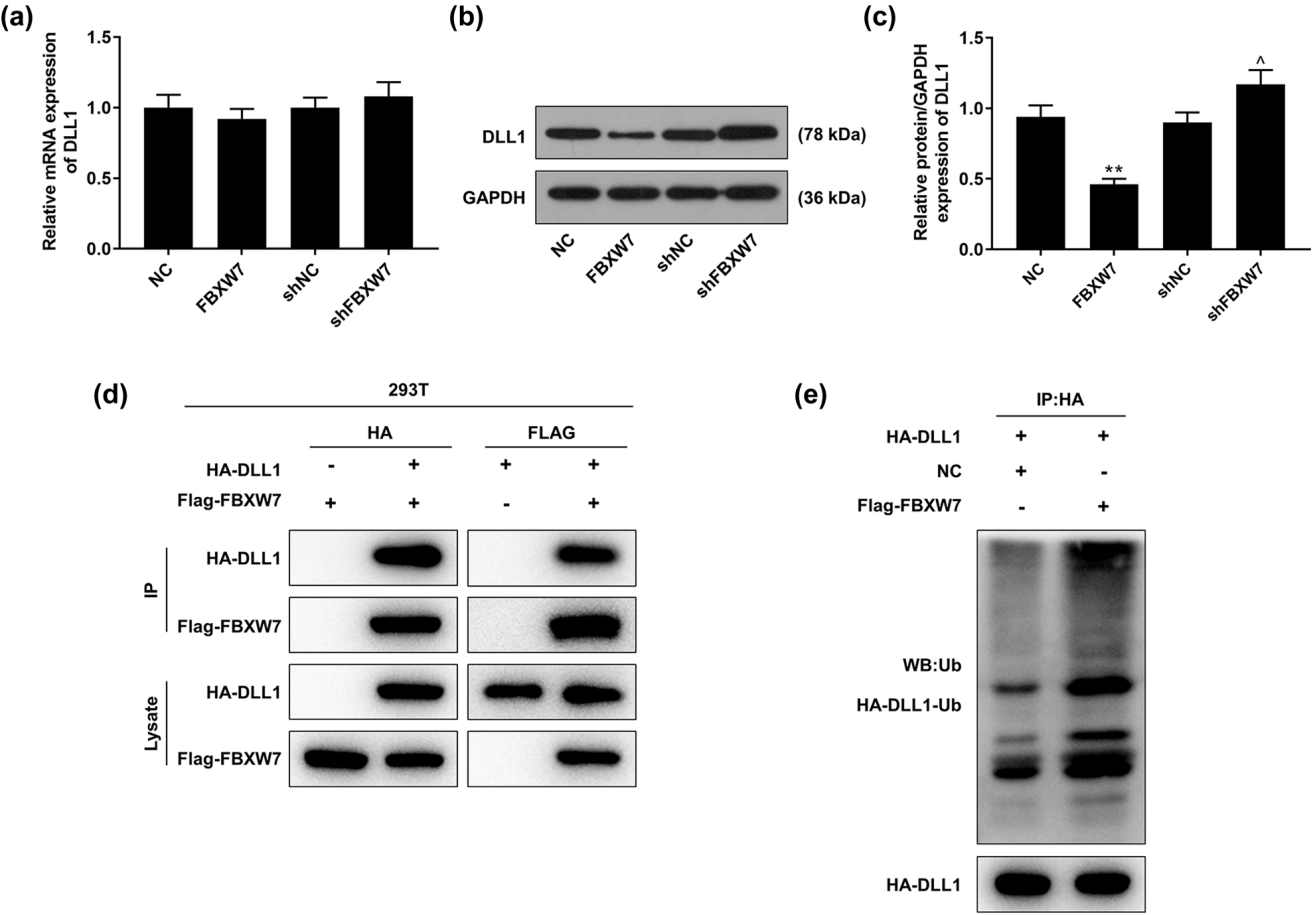
DLL1 was degraded by FBXW7 in an ubiquitin-dependent way. LX-2 cells were subjected to transfection of FBXW7 silencing/overexpression plasmid or not. The expression of DLL1 in LX-2 cells was quantified by qRT-PCR (a) and western blot (b and c). The interaction between FBXW7 and DLL1 was detected by coimmunoprecipitation (d) and western blot (e). The significant difference was evaluated by ANOVA, followed by Tukey’s *post hoc* test. ^**^ *p* < 0.001 vs NC; ^^^ *p* < 0.05 vs shNC. ANOVA, analysis of variance; DLL1, delta-like ligand 1; FBXW7, F-box and WD repeat domain containing 7; shFBXW7, short hairpin RNA against FBXW7; shNC, shRNA negative control; qRT-PCR, quantitative reverse transcription polymerase chain reaction.

As revealed in Figure 4a, qRT-PCR determined that neither FBXW7 overexpression nor FBXW7 silencing had any effect on the mRNA expression of DLL1 in LX-2 cells. However, the protein level of DLL1 in the cells was notably decreased by FBXW7 overexpression (Figure 4b and c, *p* < 0.001), but was increased by FBXW7 silencing (Figure 4b and c, *p* < 0.05). As shown in Figure 4d, DLL1 was detected to be coimmunoprecipitated with FBXW7 in 293T cells by anti-FLAG or anti-HA antibody. Of note, FBXW7 overexpression was determined to promote the ubiquitin-dependent degradation of DLL1 in 293T cells (Figure 4e).

### 3.5 DLL1 overexpression reversed the effects of FBXW7 overexpression on LX-2 cell cycle, viability, and proliferation

**Figure 5:**
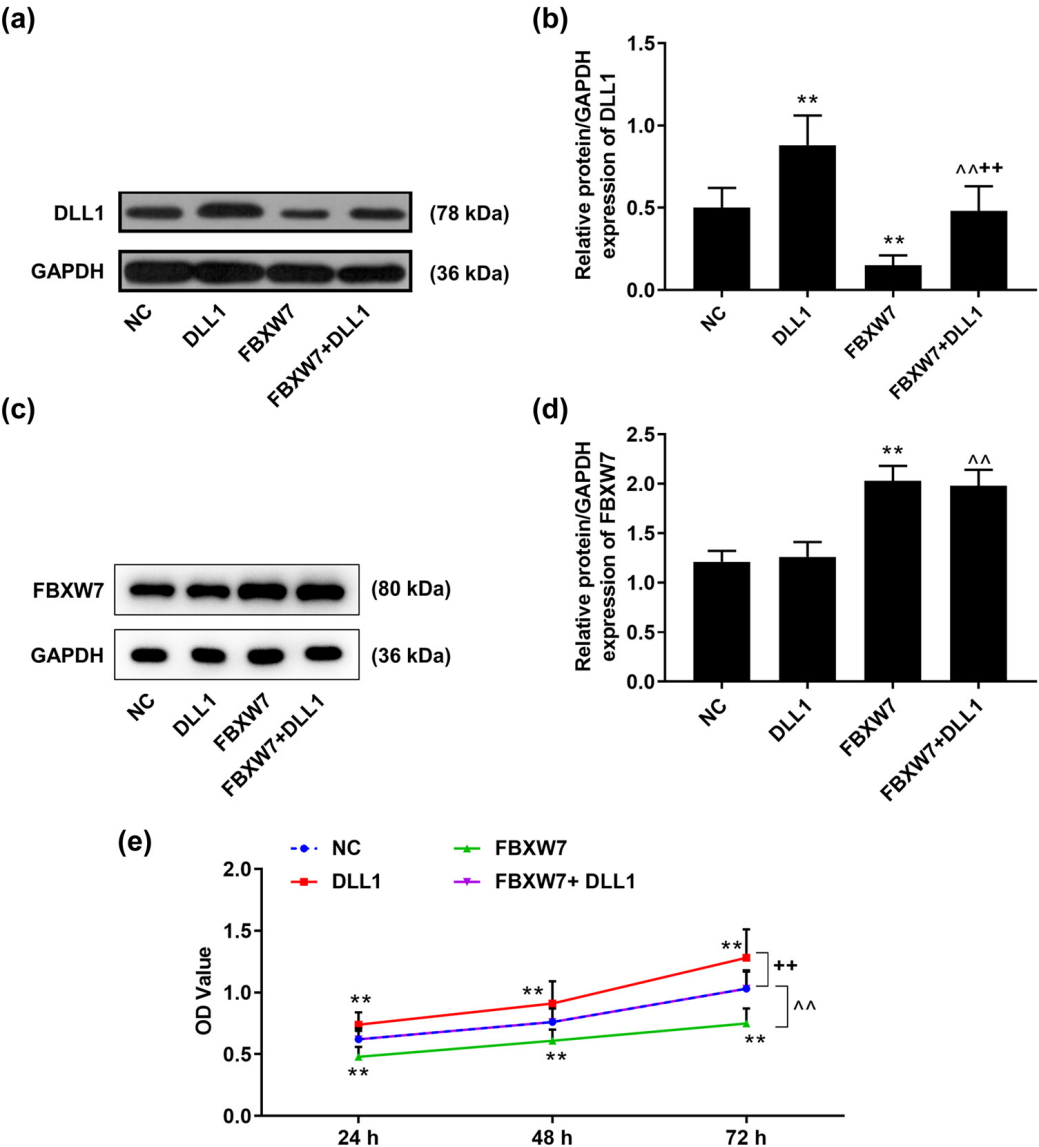
DLL1 overexpression reversed the effect of FBXW7 overexpression on LX-2 cell viability. LX-2 cells were transfected with or without FBXW7/DLL1 silencing or overexpression plasmid. Western blot was applied to detect DLL1 and FBXW7 protein levels in LX-2 cells (a–d). CCK-8 assay was performed to detect LX-2 cell viability (e). The significant difference was determined by ANOVA, followed by Tukey’s *post hoc* test. ^**^ *p* < 0.001 vs NC; ^^^^ *p* < 0.001 vs DLL1; ^++^ *p* < 0.001 vs FBXW7. CCK-8, cell counting kit-8; DLL1, delta-like ligand 1; FBXW7, F-box and WD repeat domain containing 7; NC, negative control.

**Figure 6:**
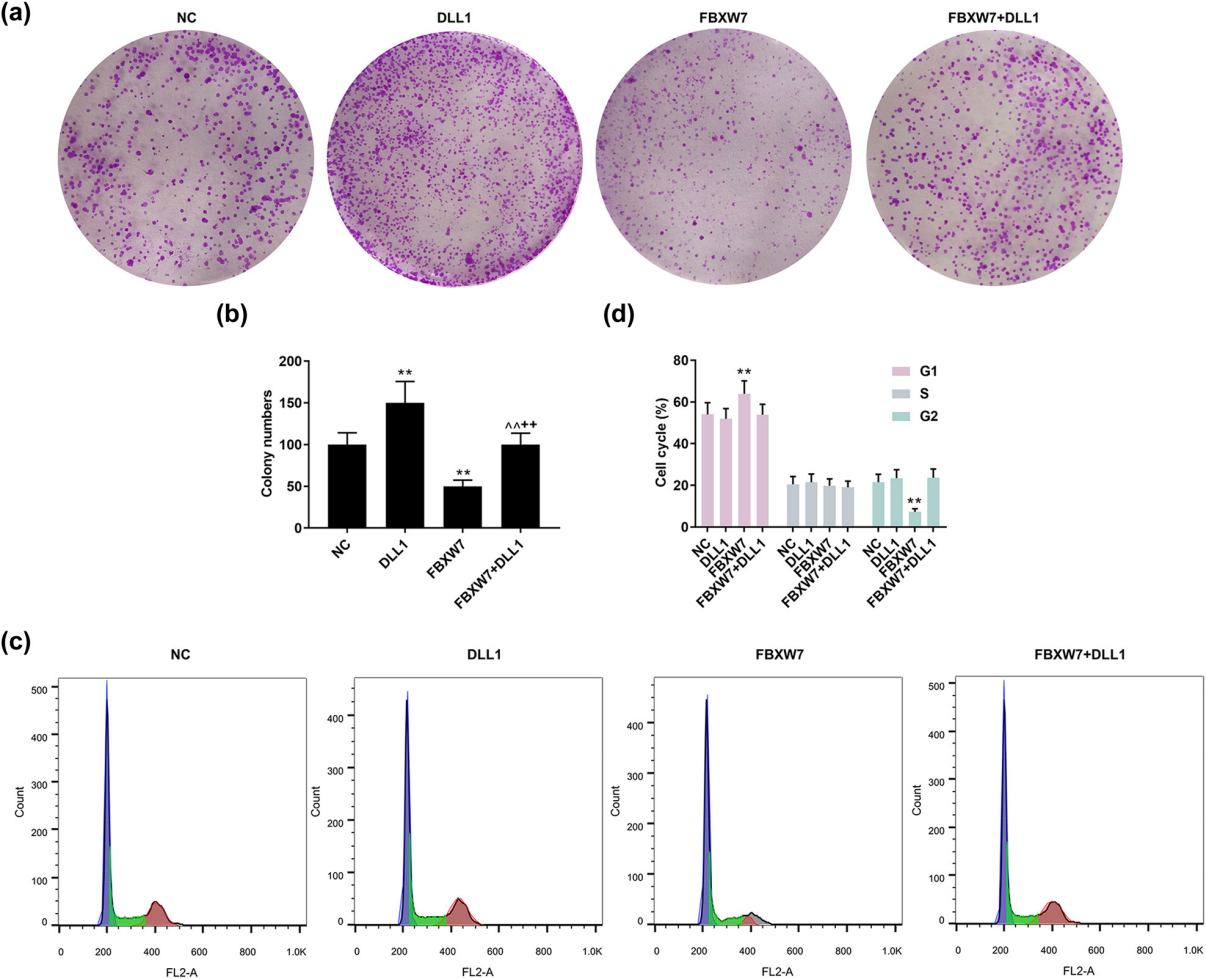
DLL1 overexpression offset the effects of FBXW7 overexpression on LX-2 cell cycle and proliferation. LX-2 cells were subjected to transfection of FBXW7/DLL1 silencing or overexpression plasmid. Colony formation assay was used to detect cell proliferation (a and b). Flow cytometry was employed to analyze cell cycle (c and d). The significant difference was detected by ANOVA, followed by Tukey’s *post hoc* test. ^**^ *p* < 0.001 vs NC; ^^^^ *p* < 0.001 vs DLL1; ^++^ *p* < 0.001 vs FBXW7. ANOVA, analysis of variance; DLL1, delta-like ligand 1; FBXW7, F-box and WD repeat domain containing 7; NC, negative control.

As illustrated in Figure 5a and b, the protein level of DLL1 in LX-2 cells was up-regulated by DLL1 overexpression plasmid (*p* < 0.001), which was counteracted in the presence of FBXW7 (*p* < 0.001). Meanwhile, the inhibiting effect of FBXW7 overexpression on DLL1 protein level was negated by DLL1 overexpression (Figure 5a and b, *p* < 0.001). However, the protein level of FBXW7 in LX-2 cells was barely affected by DLL1 overexpression (Figure 5c and d). Additionally, the results of rescue assays revealed that DLL1 overexpression enhanced the LX-2 cell viability (Figure 5e, *p* < 0.001) and proliferation (Figure 6a and b, *p* < 0.001), yet barely affected LX-2 cell cycle (Figure 6c and d). These facilitating effects of DLL1 overexpression were evidently negated by FBXW7 overexpression (Figures 5c–6b, *p* < 0.001). Furthermore, the inhibiting effects of FBXW7 overexpression on cell viability and proliferation were nullified by DLL1 overexpression (Figures 5c–6b, *p* < 0.001).

### 3.6 DLL1 overexpression offset the suppressive effects of FBXW7 overexpression on α-SMA and Collagen I expressions and Notch signaling pathway in LX-2 cells

**Figure 7:**
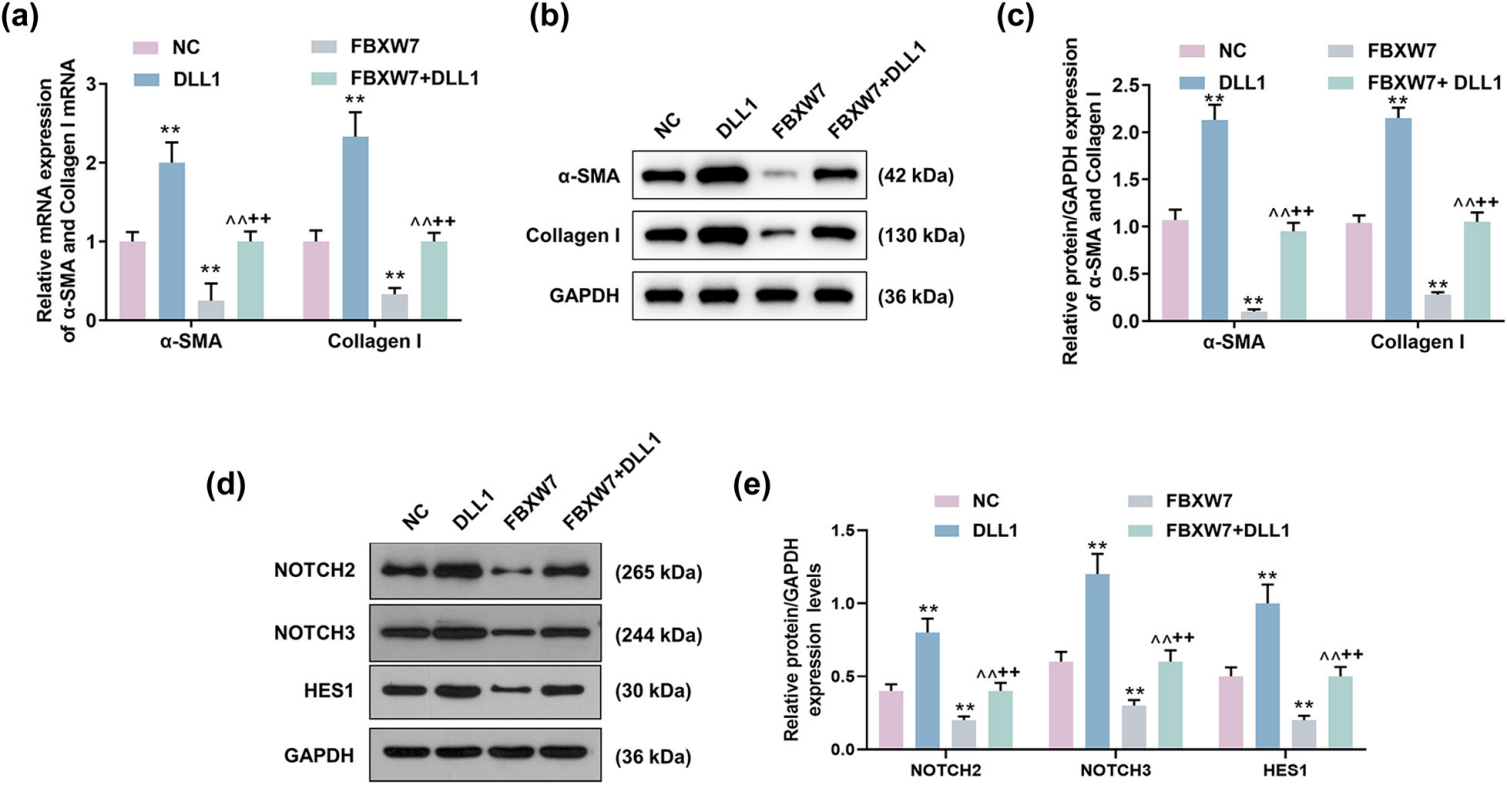
DLL1 overexpression neutralized the effect of FBXW7 overexpression on α-SMA and Collagen Ⅰ expressions and Notch signaling pathway in LX-2 cells. LX-2 cells were transfected with or without FBXW7/DLL1 silencing or overexpression plasmid. The expression levels of α-SMA and Collagen Ⅰ in LX-2 cells were measured by qRT-PCR (a) and western blot (b and c), where GAPDH was employed as the internal control. The protein levels of NOTCH2, NOTCH3, and HES1 in LX-2 cells were determined by western blot (d and e), with GAPDH serving as the internal control. The significant difference was measured by ANOVA, followed by Tukey’s *post hoc* test. ^**^ *p* < 0.001 vs NC; ^^^^ *p* < 0.001 vs DLL1; ^++^ *p* < 0.001 vs FBXW7. ANOVA, analysis of variance; DLL1, delta-like ligand 1; FBXW7, F-box and WD repeat domain containing 7; α-SMA, α-smooth muscle actin; NC, negative control; qRT-PCR, quantitative reverse transcription polymerase chain reaction.

Molecularly, DLL1 overexpression increased α-SMA and Collagen Ⅰ mRNA expressions in LX-2 cells (Figure 7a, *p* < 0.01), which was neutralized by FBXW7 overexpression (Figure 7a, *p* < 0.01). Besides, the results of western blot assay uncovered that DLL1 overexpression induced up-regulation of α-SMA and Collagen I protein levels in LX-2 cells as compared with the NC (Figure 7b and c, *p* < 0.01), but this tendency was reversed by FBXW7 overexpression (Figure 7b and c, *p* < 0.01). Likewise, down-regulations of α-SMA and Collagen I induced by FBXW7 overexpression in the cells were significantly elevated in the presence of DLL1 (Figure 7a–c, *p* < 0.001). Furthermore, DLL1 overexpression alone up-regulated NOTCH2, NOTCH3, and HES1 protein levels in LX-2 cells (Figure 7d and e, *p* < 0.001), while these tendencies were opposite when the cells were transfected with FBXW7 overexpression plasmid (Figure 7d and e, *p* < 0.001). Notably, DLL1 overexpression and FBXW7 overexpression exerted mutually reversing effects on NOTCH2, NOTCH3, and HES1 protein levels in LX-2 cells (Figure 7d and e, *p* < 0.001).

## 4 Discussion

In this study, we found that the expression of FBXW7 in LX-2 cells was negatively associated with cell cycle, viability, and proliferation according to the results of cell functional assays. It has been demonstrated that the activation of HSCs is the main cellular origin to secrete matrix proteins like Collagen I, and promote the release of pro-inflammatory cytokines in the liver tissues [26]. As the main component of ECM, Collagen I is massively deposited in the liver when HSCs are actively proliferated by injurious stimulation, which reflects the degree of HF to some extent [27]. In addition, it is commonly recognized that an enhanced expression of α-SMA in HSCs is the signal for the activation of stellate cells with a quiescent phenotype into myofibroblasts with a proliferation phenotype [28]. Through western blot, we found that both Collagen I and α-SMA protein levels in LX-2 cells were down-regulated by FBXW7 overexpression, while being up-regulated in the absence of FBXW7. Collectively, it is indicated that FBXW7 could participate in the progression of HF by regulating HSC activation.

The activation of HSCs is a dynamic and complicated process modulated by multiple signaling pathways [29]. In the study of HF, previous studies have highlighted that Notch signaling pathway is responsible for controlling the fate of HSCs, and suppressing this pathway could effectively block HSC activation [30,31]. DLL1, a Notch ligand, acts as an adhesion molecule for mast cells [32]. A previous study demonstrated that the DLL1^+^ cells were significantly expanded in diffuse mesenteric fibrosis in pristane-induced Lgals3^−/−^ mice [33]. Besides, according to several published reports, Notch ligand overexpression is found in chronic fibrosis-related diseases such as pulmonary fibrosis and cardiac fibrosis [34,35]. In this study, we predicted that FBXW7 could act as an E3 ligase for DLL1, and the regulatory effect of FBXW7 on the activation of HSCs might be realized by degrading DLL1. Indeed, this study subsequently confirmed that FBXW7 overexpression in LX-2 cells considerably reduced the protein level of DDL1, and FBXW7 silencing worked oppositely. Additionally, the ubiquitination-related mechanism of FBXW7 in HF remains unclear. Thus, we carried out ubiquitination assay, and found that there was a binding relationship between FBXW7 and DLL1. Notably, we further authenticated that FBXW7 could promote the degradation of DLL1 in an ubiquitination manner. Previously, DDL1 deficiency-induced inhibition of Notch signaling pathway was confirmed to pathologically activate liver stromal cells and promote the development of hepatic metastases [36]. On the contrary, this study first found that DDL1 overexpression facilitated the viability and proliferation of LX-2 cells as well as up-regulated Collagen I and α-SMA expressions, suggesting that DDL1 is implicated in the progression of HF by activating HSCs and promoting ECM accumulation.

As described in a previous research, the activation of Notch signaling pathway is the key factor driving the active transformation of HSCs, signifying that blocking this signaling pathway could be a therapeutic strategy to reverse HF [37]. It has been demonstrated that high expressions of Notch 2 and Notch 3 trigger the downstream gene HES1 and thereby promote the activation of HSCs [38]. Additionally, HES1 has been believed to participate in the generation of collagen [39]. Recently, the study of Zhang et al. highlighted that balancing the expression of HES1 could be an anti-fibrotic strategy against liver fibrosis [40]. Based on the pivotal roles of these three markers in the Notch signaling pathway, we further performed western blot to investigate the regulatory effect of FBXW7 on this pathway in LX-2 cells. As a result, we observed that FBXW7 overexpression prominently down-regulated NOTCH2, NOTCH3, and HES1 levels, whereas DLL1 overexpression induced elevated protein levels of these three genes. Moreover, DLL1 overexpression and FBXW7 overexpression had mutually reversing effects on NOTCH2, NOTCH3, and HES1 protein levels in LX-2 cells. Taken together, it is indicated that FBXW7 plays an inhibitory role in the active transformation of HSCs by reducing DLL1 expression to block the Notch signaling pathway.

There are some limitations in the current study, such as the lack of animal and human studies. Although the findings of this study could be the basis of future animal experiments and human trials, further investigations with animal model and clinical samples are necessary in the future.

In conclusion, our study provides the basic evidence that FBXW7 overexpression inhibits the progression of HF *in vitro*. Mechanistically, miR-497 overexpression ubiquitination-dependently degrades DLL1 to block the Notch signaling pathway, thereby repressing the activation of HSCs and ECM accumulation. Hence, targeting miR-497 might be a potential therapeutic strategy for HF.
